# Atypical thyroid manifestation in Cowden disease: a case report and literature review

**DOI:** 10.3389/fped.2025.1499664

**Published:** 2025-03-03

**Authors:** Marion Garcia, Isabelle Oliver Petit, Camille Franchet, Olivier Abbo, Audrey Cartault, Frédérique Savagner

**Affiliations:** ^1^ Biochemistry and Genetic Laboratory, Federative Institute of Biology, CHU, Toulouse, France; ^2^ Biochemistry Laboratory, University Paul Sabatier, Toulouse, France; ^3^ Endocrine, Genetics, Bone Diseases, and Paediatric Gynecology Unit, Children’s Hospital, CHU, Toulouse, France; ^4^ Pathology Department, Oncopole Claudius Regaud, Toulouse, France; ^5^ Pediatric Surgery Department, Children’s Hospital of Toulouse, CHU, Toulouse, France; ^6^ Inserm UMR1297, Team 9, Toulouse, France

**Keywords:** Cowden syndrome, childhood, thyroid pathologies, *PTEN* variant, hyperfunctioning nodule

## Abstract

**Background:**

Cowden syndrome (CS) is a complex and rare hereditary disorder characterized by a high risk of developing both benign and malignant tumors. Germline variants in the *PTEN* gene lead to this autosomal dominant syndrome, which predisposes individuals to lesions of the skin and mucous membranes, as well as breast, thyroid, endometrial, and kidney cancers. Early identification of symptoms is essential for implementing effective therapeutic strategies, especially in managing thyroid cancer risk.

**Case presentation:**

During a tonsillectomy in an 8-year-old boy, the surgeon incidentally noted a left lateralized thyroid swelling. The clinical picture of Cowden syndrome was further supported by the presence of macrocephaly and intellectual disability since birth along with rare and atypical thyroid disorder marked by a toxic adenoma. Genetic analysis of both the tissue and blood samples confirmed the diagnosis. The clinical manifestation of thyroid issues in a young child may indicate CS, a condition that is often poorly assessed by clinicians. Family history revealed that the boy's father and sister also carry the same heterozygous variant, presenting a spectrum of Cowden syndrome manifestations.

**Conclusion:**

Molecular analysis of the *PTEN* gene should be considered in young patients with thyroid nodules or nodules associated with abnormal thyroid function test, even without clear evidence of Cowden syndrome, particularly if there is a family history of thyroid, breast, or hamartoma-related conditions.

## Introduction

Cowden syndrome (CS) is a rare genetic disease affecting 1/200,000 to 1/250,000 people worldwide transmitted through an autosomal dominant mode (1). This genodermatosis affecting both infants and adults is characterized by multiple hamartomas and a high-risk predisposition to cancer. It is associated to germline loss of function variants in the *PTEN* gene as a tumor suppressor regulating cell growth and division through the P13K/AKT/mTOR signaling pathway (2). The Cowden syndrome was first described in 1963 by Lloyd and Dennis in a 20-year-old patient named Rachel Cowden who was diagnosed with thyroid, breast and oral mucosa lesions (3, 4). In childhood, presentation includes macrocephaly for nearly 95% of cases, as a major criteria defined by the National Comprehensive Cancer Network (NCCN) (5–7). Other minor criteria such as intellectual disability, lipomas, thyroid goiter may point to this diagnosis when two of them were combined, especially for affected relatives without macrocephaly. Benign thyroid lesions are the most common minor criterion (75% of cases) while thyroid cancer affects approximatively 24% of all CS patients (8). In adults, thyroid lesions predominantly manifest in women at a younger age compared to sporadic nodules whereas no correlation with gender or puberty has been noted for children and adolescents (9, 10). Notably, children with CS exhibit significant thyroid involvement with up to 50% diagnosed with benign lesions at a mean age of 9 years and a incidence of 4%–12%, mainly of the follicular pattern (8). Thyroid hormone profiles are generally normal without anti-thyroid antibodies although hormonal dysfunction may arise if thyroid nodules are diagnosed late (10). To date, if goiter and some grave's diseases has been described in CS, only one case of non-autoimmune hyperthyroidism has been documented in the literature (12–14).

Due to the risk of thyroid cancer, thyroidectomy should be considered for PTEN-positive patients upon detection of thyroid nodules, despite no discernible difference in the natural history of thyroid cancer (1, 11). A benign thyroid disorder can progress to a malignant tumor in the presence of somatic cancer driver genes as TSHR for hyperfunctioning nodules, with the risk for cancer identified only through aggressive histological features (15–17). It is particularly true for adults whereas for children, thyroidectomy should be considered depending on the size of the nodule and the location of the mutation (18). Our case underscores the importance of early CS diagnosis that may initially manifest as a simple thyroid dysfunction in childhood but present a large thyroid nodule with a risk of cancer development pressing early thyroidectomy. Recent comprehensive guidelines advocate for surveillance starting at age 18 for thyroid cancer risk in pediatric patients with CS and susceptibility to thyroid disorders (11). However, additional research is needed to improve clinical outcomes for affected individuals and understand the global risk of carcinogenesis.

## Case report

**Figure 1:**
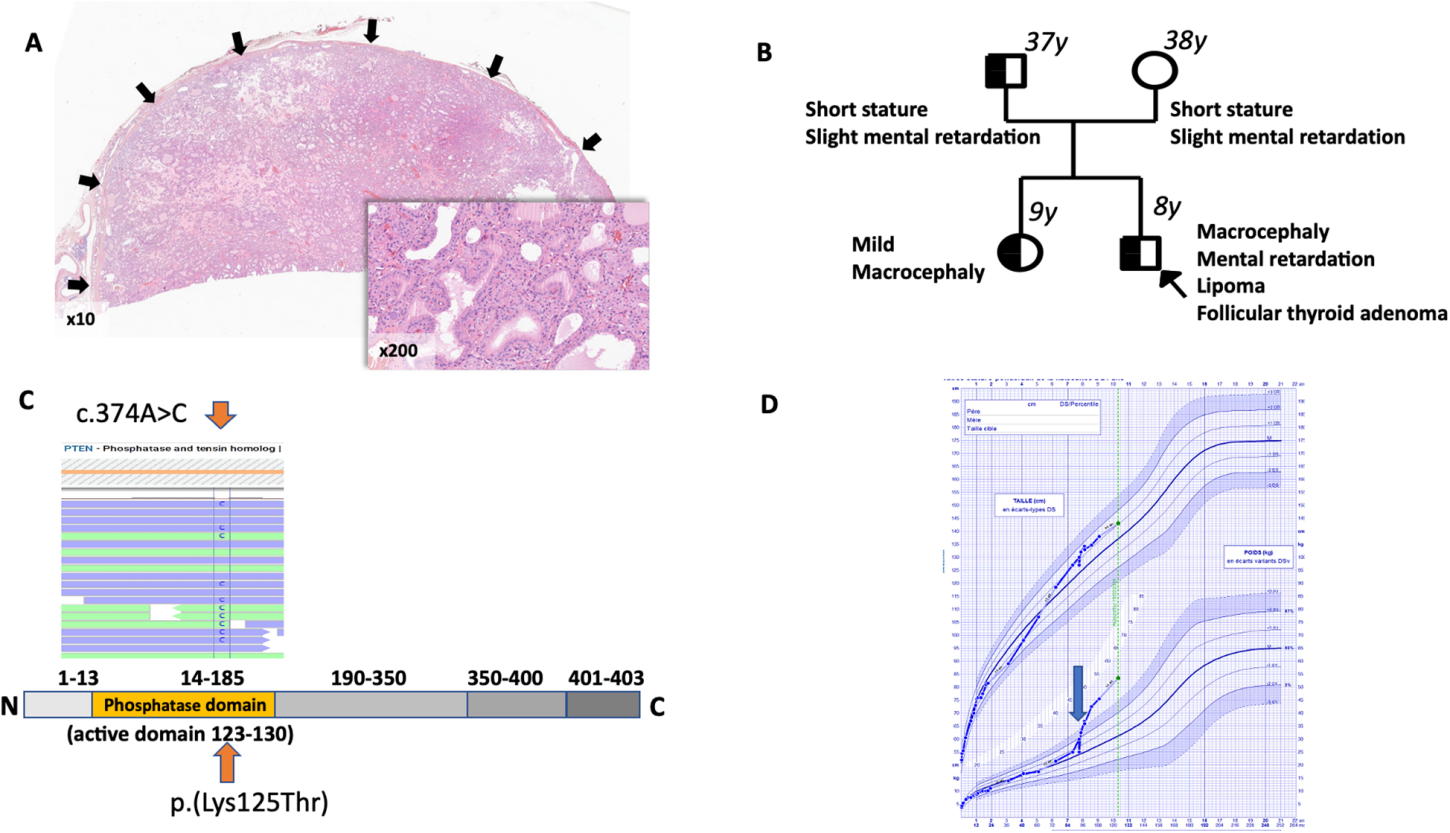
**(A)** histologic examination of thyroid tumor *(magnification ×10)*: follicular adenoma is fully delimitated by a thin capsule (arrows) with neither capsular nor vascular invasion. *Insert (magnification ×200)*: This nodule is being characterized by a normo- and macrofollicular growth pattern with no nuclear features of papillary thyroid carcinoma. **(B)** Localization of the *PTEN* variant. Germline heterozygous variant c.374A>C was detected (orange arrow) by Next Generation Sequencing (Illumina, custom thyroid cancer panel) in exon 5 of the *PTEN* gene (LRG_492, NM_177438.3); The sequencing depth was 874× and 786× respectively for blood (allele frequency 49.8%) and tissue (allele frequency 74.4%) samples (Integrative Genomic Viewer Software). No additional *PTEN* variants were identified at the somatic level. **(C)** Family pedigree. Individuals screened for *PTEN* germline variant are indicated by a black border with age at molecular diagnosis for each patient explored. Heterozygous positive variants are indicated by a black filling. Clinical phenotypes are associated to each patient. The proband is marked with an arrow. **(D)** Growth curve of the male child. At 8 years old growth increased (+4.7 SD in weight) was noted (blue arrow).

An 8-year-old boy, placed in foster care at birth, was referred to a surgeon due to severe sleep apnea syndrome persisting for 2 years. During the tonsil removal, a left-sided cervical swelling in the thyroid region was discovered, confirmed by ultrasound to be a isthmic 45 mm thyroid nodule EU-TIRADS 3 with a 2%–4% risk of malignancy (19). Despite the current recommendations (20), thyroid scintigraphy was not performed as first line ultrasound imaging identifying a large nodule was considered sufficient. Fine-needle aspiration cytology classified the nodule as benign follicular features [Bethesda class II (21),]. Definitive histology following thyroid surgery confirmed a large follicular adenoma (Figure 1A). Indeed, the thyroid gland contained a solitary tumor, well delimitated by a thin capsule, with no evidence of capsular or vascular invasion. The nodule was composed of normofollicular and macrofollicular areas. Cytologically, no nuclear features of papillary thyroid carcinoma appeared. Next Generation Sequencing was secondarily performed on the nodule and an alteration in *PTEN* gene appeared, corresponding to the patient's germline mutation [i.e., p.(Lys125Thr) with a variant allele frequency of 74.4%]. Moreover, no additional pathogenic variant neither in *RAF and RAS* nor in *TSHR* and *GNAS1* genes was shown.

The foster family had not reported particular issues in childhood. However, two months prior to the tonsil surgery, clinical signs of hyperthyroidism caused by the hypersecreting nodule were retrospectively identified. These included heavy sweating, tremors and nervousness which were consistent with the hormonal assessment conducted after tonsil surgery: TSH of 0.01 IU/ml (reference range: 0.27–0.42 IU/ml), FT4 of 16.1 pg/ml (reference range: 9.3–17 pg/ml), FT3 of 6 pg/ml (reference range: 2–4.4) and negative TSHR and TPO antibodies. Calcitonin level was not measured. Pharmacological treatment with methimazole at 0.5 mg/kg per day for 6 months, achieved balanced euthyroidism within one month. Clinical investigations revealed primary nonprogressive macrocephaly (+2SD) associated with hydramnios at birth, mild axial hypotonia, strabismus, minimal cutaneous manifestations and slight intellectual disability consistent with NCCN criteria for CS diagnosis (5).

Due to foster care status for the proband and his sister, biological parents were subsequently evaluated. Both non-consanguineous parents exhibited mild intellectual disability and short stature. At the age of 36, the father has a head circumference of 60.1 cm (reference range: 52.5–58.5 cm) and presents with spondylolisthesis which could retrospectively be associated with PTEN-related vertebral hemangiomas (22). His sister displayed mild macrocephaly (+1SD) and tall stature and no neurodevelopmental delay. All patients have signed an informed consent. Genetic analysis via CGH-array was negative for both children. Next generation sequencing of a familial thyroid cancer gene panel (see Supplementary Table 1) revealed a heterozygous missense variant c.374A>C in exon 5 of the *PTEN* gene (NM_000314) resulting in the substitution of a basic lysine residue with an hydroxylic threonine residue p.(Lys125Thr) confirmed by sanger analysis (Figure 1B). This variant in the phosphatase tensin domain of the protein was referred as likely pathogenic (class 4) in LOVD and using missense predictive software (GeneBe, SIFT, Polyphen-2, Franklin, AlphaMissense, Revel and Clinpred) according to the American College of Medical Genetics classification. It was located in a hotspot region for mutations that abrogate PTEN's phosphatase activity and was not currently referenced in gnomAD exome or Clinvar databases (18). Molecular analysis confirmed the presence of this variant in his sister and father but not in his mother (Figure 1C). However, all relatives have had normal thyroid ultrasounds and TSH levels so far.

Given the size of the thyroid nodule and the presence of hyperthyroidism, a total thyroidectomy was performed 6 months after fine-needle aspiration cytology. Euthyroidism was achieved with a daily L-T4 dose of 112 µg. Hyperthyroidism was primarily caused by the delayed management of the thyroid mass. Due to euthyroidism following surgery and increased food requirements related to CS, a deviation in the growth curve was observed, with a +4.7 SD in weight (Figure 1D) associated to +1SD in height.

## Discussion

The incidental discovery of a benign thyroid lesion associated with hyperthyroidism in this 8-year-old boy has significantly impacted the medical care and surveillance of this index case within a Cowden syndrome family. Hyperthyroidism or toxic adenoma in CS have been described very rarely in the literature to date (14). Just as rarely, medullary thyroid cancer can coexist with hyperfunctioning goiter (23). However, in our case, macrocephaly did not warrant measuring calcitonin. In our case, normalization of thyroid hormone levels resulted in a significant shift in the growth curve in relation to metabolic syndrome and obesity observed for CS, despite insulin sensitivity (24). We postulate that hyperthyroidism may have mitigated the typical weight gain observed in CS patients during childhood (25). Euthyroidism obtained after thyroid surgery subsequently revealed an increase in adiposity, linked to PTEN-enhanced insulin signaling in the muscle and liver, as previously reported (24).

A total thyroidectomy has been recommended relative to the large thyroid nodule identified and the potential risk of thyroid cancer progression during adolescence (26). Recommendations for thyroid cancer surveillance vary among expert panels with a consensus on annual monitoring through neck palpation, thyroid ultrasound and serum TSH measurement starting at age 18 or 5–10 years before the age of the first known thyroid cancer case in the family (5, 11). However, ultrasound surveillance could be adapted based on initial results, especially to limit ultrasounds in children and adolescents (27). Children with PTEN-related syndromes are rarely at risk of developing hyperthyroidism except in case of concomitant Graves’ disease, as referred in the literature (13). Regular thyroid function monitoring is crucial for individuals with *PTEN* variants to rapidly manage any related issues. Recently, pathogenic variants in exon 5 of the *PTEN* gene have been associated with an increased cancer risk, prompting the consideration of starting surveillance from age 10 (28). Exon 5 encodes the core catalytic domain of the PTEN protein (active site between amino acid 123 and 130) and resulting mutations disrupt the pan-phosphatase (lipid and protein) activity that regulates the PI3K cascade (29, 30). Given the familial variant's location in exon 5, the boy's sister, who currently shown no nodules, undergoes annual follow-up.

Considering the prevalence of somatic *PTEN* variants in follicular adenomas and mostly benign thyroid lesions in CS, *PTEN* mutations may play a role in early neoplastic follicular cell growth that could explain the frequency of thyroid pathologies in CS (31, 32). Furthermore, the presence of follicular adenomas in children and young adults should raise suspicion of inherited conditions, primarily CS or DICER1 syndrome, necessitating family screening (33).

The risk for developing thyroid cancer as well as breast, kidney, endometrial and colorectal cancer is increased in both PTEN-positive adults and children, with the risk being higher in adults (26). The risk for developing other types of cancer remains comparable to that of the general population (34). The NCCN (v.2021) has established guidelines for surveillance once a *PTEN* gene mutation is identified, regardless of its location within the gene. This includes comprehensive clinical examination starting at age 18 or earlier if there is a cancer diagnosis in the family. Colonoscopy from age 35 and then every 5 years should be considered if symptomatic or if polyps are detected. Renal ultrasound from age 40, repeated every 1–2 years, can aid in early cancer detection. Dermatological, psychomotor and dental examinations may also be integrated into the surveillance protocol. Patient education on recognizing signs and symptoms of malignant tumor development is crucial due to the increased carcinogenic risk associated with the syndrome. Management strategies must be tailored accordingly, distinguishing this approach from that used for the general population.

In conclusion, the case of this 8-year-old boy illustrates an incidental finding of CS based on unusual hyperthyroidism and a challenging family history relative to foster cares. Multidisciplinary care and personalized medical attention are essential for managing the risk of malignant progression in this syndrome.

## Data Availability

The datasets presented in this study can be found in online repositories. The names of the repository/repositories and accession number(s) can be found in the article/Supplementary Material.
